# Getting Good Sleep with Family Support: The Role of Fear of Crime and Loneliness

**DOI:** 10.3390/bs13110909

**Published:** 2023-11-07

**Authors:** Chun Xia, Jia Xu, Yaya Wang

**Affiliations:** 1 School of Educational Science, Anhui Normal University, Jiuhua-Nan-Road 189, Wuhu 241000, China; xiachun@mail.ahnu.edu.cn; 2 School of Marxism, Anhui Normal University, Jiuhua-Nan-Road 189, Wuhu 241000, China; xujia0550@ahnu.edu.cn; 3 School of Finance, Taxation and Public Administration, Tongling University, Cuihu-Si-Road 1335, Tongling 244061, China

**Keywords:** family support, sleep quality, fear of crime, loneliness, gender

## Abstract

Sleep problems in middle-aged and older people can threaten their physical and mental health. Family support is regarded as a key factor that affects sleep quality, but the influence mechanism remains underexplored. This study analyzes the mediating effects of fear of crime (FOC) and loneliness in the relationship between family support and sleep quality, and explores whether gender plays a moderating role between family support and FOC. A questionnaire survey was conducted among 1043 Chinese middle-aged and older people aged 45–93 years. Using 10,000 bootstrapped samples, the study shows that middle-aged and older people who receive more family support have better sleep quality, and FOC and loneliness play mediating role in this association. Gender moderates the relationship between family support and FOC. Compared with men, family support for females has a greater impact on their FOC condition, and the mediating effect of family support on sleep quality through FOC is also greater among women. Family support can affect sleep quality through the chain mediating effect of FOC and loneliness for women. This study provides an in-depth understanding of the relationship between family support and sleep quality.

## 1. Introduction

Suboptimal sleep is a key issue faced by middle-aged and older people globally [1,2,3]. Problematic sleep not only leads to physical problems but also psychological problems, affecting people’s working status and overall life satisfaction [4,5,6,7]. Scholars in the field of medicine, psychology, sociology, management, and many other disciplines have studied sleep quality as a common concern [8,9,10]. Enhancing social support, especially support from family members and intimate relatives, can improve the sleep quality of middle-aged and older people [1,3,11,12,13]. Several researchers have found a robust positive correlation between social support and sleep quality [14,15,16,17]. However, the systematic mechanism of the impact of family support on the sleep quality of middle-aged and older people remains understudied [9,18,19]. To address this research gap, this study proposes a regulated chain mediating effect model to explore the in-depth value of family support in improving the sleep quality of middle-aged and older people. In addition to clarifying this impact mechanism, this study also investigates an innovative impact factor, fear of crime (FOC), which plays a potential mediating role in the association between family support and sleep quality.

### 1.1. Family Support and Sleep Quality

**Figure 1:**
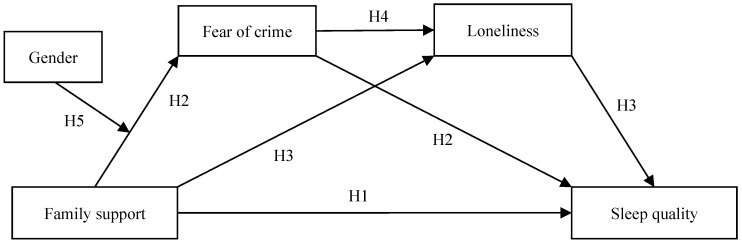
The proposed theoretical model.

Family is a core part of a social support system [18,20,21,22], and it plays a critical role in an individual’s growth and development [23,24,25,26]. In particular, family support is positively associated with sleep quality in middle-aged and older people [1,3,16]. According to the buffering model [14,18,27], families can support individuals coping with stress and reduce the adverse effects of stress on the individual, including sleep quality. In non-stressful situations, based on the social support model [14,28,29], family support aids individual development by providing a relatively safe environment that encourages career development, physical and mental health, and sleep quality [19,30,31,32,33]. A meta-analysis of 61 studies (involving 105,437 participants) found that social support had a positive impact on sleep quality, including self-reported sleep quality as well as objective assessments (e.g., actigraphy), regardless of whether people received or perceived social support [15,34,35]. Thus, we propose Hypothesis 1 of this study (shown in Figure 1).

**Hypothesis** **1.** *Family support has a positive effect on the sleep quality of middle-aged and older people.*

### 1.2. The Mediating Role of FOC between Family Support and Sleep Quality

Society and family support have a significant impact on people’s FOC [14,28,36]. On the basis of the social support model [14,28,29], support from the family could help individuals to obtain more material resources, social resources, and information resources, etc., which would allow them to have a safe environment and self-protection measures [37,38,39,40]. Further, family support would enable individuals to avoid risky behaviors to obtain basic living resources [41,42,43]. Therefore, individuals may experience lower FOC with family support [36,44]. Furthermore, FOC has a significant negative impact on people’s health [45,46,47,48] and may result in individuals experiencing high levels of anxiety and depression, and presenting avoidance behavior and sleep problems [49,50,51]. Furthermore, FOC, as a social-psychological process centered on emotion, may increase the secretion of epinephrine and cortisol by activating people’s stress-coping system, which could affect sleep quality [52,53]. An analysis of cross-national data of 39,590 participants from Mexico, Ghana, South Africa, India, China, and Russia using data from the World Health Organization’s Longitudinal Study on Global Aging and Adult Health (2007–2010) supports these outcomes [54]. Thus, we propose the second hypothesis (also shown in Figure 1).

**Hypothesis** **2.** *FOC plays a mediating role between family support and sleep quality.*

### 1.3. The Mediating Role of Loneliness between Family Support and Sleep Quality

Loneliness has a significant negative impact on people’s health and well-being [55,56,57,58]. Based on the social support model [14,28,29], family support is one of the most important ways to support people coping with loneliness [59,60,61,62]. Loneliness has a substantial impact on people’s health and can lead to cardiovascular disease, alcoholism, dementia, and immune system diseases, all of which can result in an increased risk of sleep problems [63,64]. The literature supports the notion that loneliness intervenes in sleep efficiency [65]; thus, lonely people have more sleep problems [4,13] and poor sleep quality [15,66]. Loneliness could also be an intermediary variable between interpersonal relationship variables (such as interpersonal stress) and sleep quality [67]. Thus, we propose the third hypothesis (also shown in Figure 1).

**Hypothesis** **3.** *Loneliness plays a mediating role between family support and sleep quality.*

### 1.4. The Chain Mediating Effect of FOC and Loneliness

We argue that the two mediator variables in this study (FOC and loneliness) have an important association with each other, with FOC affecting loneliness [49,55]. First, FOC weakens people’s interpersonal trust, resulting in an inclination to distrust others [68]. Such distrust could make it harder for people to establish healthy and stable interpersonal relationships in their social communication network, increasing the feeling of loneliness [69]. Second, FOC may lead to people having a strong tendency for avoidance behavior, which may reduce their contact and communication with people around them and make them feel alone [70,71,72,73]. It was found that the higher the FOC of older people, the stronger the loneliness they may experience in a study of 1266 low-income older people in Singapore [74]. Thus, both FOC and loneliness could be regarded as intermediary variables between family support and sleep quality, with FOC affecting the degree of loneliness. Based on the social support model [14,28,29], we propose the fourth hypothesis (also shown in Figure 1).

**Hypothesis** **4.** *Both FOC and loneliness play a chain mediating role on the association between family support and sleep quality.*

### 1.5. Regulatory Role of Gender

The effect of gender is a significant factor in research involving family support and FOC [75,76,77]. The fear-victimization paradox refers to the phenomenon that women are less likely to be victims of crime than men, but generally have a higher FOC than men [78]. Gender plays a moderating role between family support and FOC [79,80]. On the basis of the vulnerability hypothesis [81,82,83,84,85], compared with men, women’s family support has a greater impact on their FOC. First, in terms of evolution, men and women have different roles in the society [86]; men are more likely to face risks and fight to obtain their own social status, whereas women’s priority is to protect their offspring [87]. Therefore, women need more family support, and family support has a greater impact on women’s FOC than men [88]. Second, according to the vulnerability hypothesis [81,82,83,84,85], women face more difficulties in protecting themselves than men because their physical condition is generally weaker [89]. Therefore, family support is more important for women to avoid being attacked by criminals and to reduce their FOC [36]. Third, from the perspective of gender identity, men are more likely to consider themselves as protectors, while women more often identify themselves as being protected [90,91]. However, family support, particularly family support from female family members to men, conflicts with men’s general gender identity (as protectors) [86,92]. Therefore, family support has a relatively weaker impact on men’s FOC than women’s. Thus, we put forward the fifth hypothesis (also shown in Figure 1).

**Hypothesis** **5.** *Gender moderates the effect of family support on FOC. This effect is stronger in women than for men.*

## 2. Materials and Methods

### 2.1. Sample and Procedure

Participants were from Anhui Province in eastern China. Specifically, they were from a city in the east and a city in the west of Anhui, and we used a multistage sampling method to select participants. First, two counties (districts) were randomly selected from two cities, and 6 communities were randomly selected from each county (district) for interviews. We selected 100 households to survey in each community, and selected older people who had no difficulties in communicating as respondents. More specific selection criteria for respondents were (1) aged 45 years or above; (2) living in the community for at least 6 months during the past year; (3) able to communicate with the interviewers, and (4) willing to participate in our interview and sign the informed consent form after understanding the purpose of the study. This study has been approved by the ethics committee of the first author’s institution (approval number: AHNU-ET2022072). Our interviews were pen-and-paper questionnaires, and the interviewers were experienced university students. Most of the interviewees filled out the questionnaires by themselves. Given respondents’ low education level or inconvenience in filling the questionnaire, a small number of interviewees responded orally to all the questions while the interviewers filled in the questionnaire. Each interviewee received gifts worth approximately USD 2.50 after completing the questionnaire.

A total of 1200 questionnaires were distributed and 1076 were returned. We removed 33 invalid questionnaires, and 1043 valid questionnaires were analyzed. The age of the respondents was between 45 and 93 years, with an average age of 64.87 years (SD = 11.52). Male respondents were 583, accounting for 55.89% participants. The education level of all respondents was low; 515 respondents (49.38%) had junior high school education or above; 492 respondents (47.17%) had only received primary education or had no education background; 3.45% of respondents did not report their education level. There were 935 married respondents (89.65%), 96 respondents were unmarried or divorced (9.20%), while 12 respondents did not report their marital status (1.15%).

### 2.2. Measures

#### 2.2.1. Family Support

This study used 4 items of the family dimension from the Multidimensional Scale of Perceived Social Support, compiled by Zimet et al. [93], to measure the respondents’ family support, including “My family really tries to help me”, “I can get emotional help and support I need from my family”, “I can talk about my problems with my family”, and “My family is willing to help me make decisions”. Cronbach’s α coefficient in this study was 0.91. The average value of the 4 items was used as an indicator of the family support. The higher the score, the higher the family support level.

#### 2.2.2. Fear of Crime (FOC)

Referring to similar research outcomes [94], this study used 2 items to measure the FOC: whether the respondents worried about being violated by crime when they walked near their residence in the dark, and whether they were worried when they were alone at home. The responses were indicated on a 5-point Likert scale. The Cronbach’s α coefficient in this study was 0.89. This study takes the average score of these 2 items as an indicator of FOC. The higher the score, the stronger the FOC.

#### 2.2.3. Loneliness

Referring to the experience of the International Social Survey Program (ISSP), this study uses 3 items to measure loneliness according to the UCLA Loneliness Scale (Version 3) compiled by Russell [95]: how far the respondents experienced “lack of companionship”, “being neglected,” and “being isolated”. The responses were indicated on a 5-point Likert scale (from 1 = “never” to 5 = “very often”). The Cronbach’s α coefficient in this study was 0.87. The average score of the 3 items was used as an indicator of loneliness. The higher the score, the stronger the loneliness.

#### 2.2.4. Sleep Quality

This study used the method of Allen et al. [96], and the respondents were asked “How would you rate your overall sleep quality during the past month?” A 7-point Likert scale was used (from 1 = “very bad” to 7 = “excellent”), with higher scores indicating better sleep quality [53,54]. This single-item measurement has been proven to have high reliability and validity [47,97].

#### 2.2.5. Control Variables

Based on the method recommended by Bernerth and Aguinis [98], this study collected data on respondents’ age, education level (1 = junior high school and above, 0 = primary school and below), marital status (1 = married, 0 = other), and whether they lived alone or not (1 = living alone, 0 = not living alone). The scale developed by Griskevicius et al. was used to measure the socioeconomic status (SES) of respondents [99]. The scale contains 6 items and uses a 7-point Likert scale for responses (from 1 = strongly disagree to 7 = strongly agree). This scale has high reliability and validity and is widely used in SES-related research (e.g., [100]). In this study, the Cronbach’s α coefficient was 0.84, and the mean value of the scale items was used as the indicator of SES. The higher the score, the higher the SES.

### 2.3. Data Analysis

This study uses SPSS 25.0 and M-plus 7.6 for data analysis. We first analyzed the direct impact of family support on sleep quality. Further, the chain mediated effect of FOC and loneliness in this association was analyzed, while exploring the moderating role of gender between family support and FOC [101]. Before constructing our model, we performed mean-centered continuous variable analysis [102]. This study set the number of repeated samples to 10,000 when using bootstrap for mediation and moderation effect analysis. Control variables such as gender, age, marital status, and SES were added in the model during the analysis unless exclusions of any control variables are specified in the text.

## 3. Results

### 3.1. Results of Descriptive Statistical Analysis

The mean, standard deviation, and correlation coefficient between variables are shown in Table 1. Family support for middle-aged and older people was significantly positively correlated with their sleep quality, but significantly negatively correlated with FOC and loneliness, while FOC and loneliness were significantly negatively correlated.

### 3.2. Common Method Bias Test

As this study uses cross-sectional data, there might be a risk of common method bias when the same group is measured [103]. Therefore, we used Harman’s single factor test to check the data [104]. The results show that seven factors out of all items in the variables could be extracted using exploratory factor analysis, but the factor with the largest explanatory rate could only explain 20.427% of the total variance, which was far lower than the recommended explanatory rate criteria [104,105]. Therefore, our data did not suffer from severe common method bias.

### 3.3. The Direct Impact of Family Support on Sleep Quality

Family support had a significant positive impact on sleep quality (B = 0.126, *p* = 0.001); respondents who received more family support had better sleep quality. The bootstrap analysis also confirmed that family support had a significant positive effect on sleep quality (95% confidence interval (CI) = [0.051, 0.201] of 10,000 bootstrap sampling calculations [does not contain zero]). Consequently, Hypothesis 1 was supported.

### 3.4. Family Support Affects Sleep Quality through FOC and Loneliness

We examined three pathways: (1) family support affects sleep quality with respect to FOC, (2) family support affects people’s sleep quality with respect to loneliness, and (3) the chain mediation effect of the family-support–FOC–loneliness–sleep-quality pathway. Family support had a significant negative impact on FOC, while FOC had a significant negative impact on sleep quality. The impact of family support on sleep quality with respect to FOC was also significant, with 95% CI not containing 0, indicating that FOC was a mediator variable between family support and sleep quality. Family support also had a significant negative impact on loneliness, loneliness had a significant negative impact on sleep quality, and the mediation effect of family support on sleep quality with respect to loneliness was also significant. The chain mediation effect of family support on FOC, and from FOC on sleep quality with respect to loneliness was also significant. Therefore, Hypotheses 2, 3, and 4 were all supported. The results are shown in Table 2.

### 3.5. Moderating Effect of Gender

**Figure 2:**
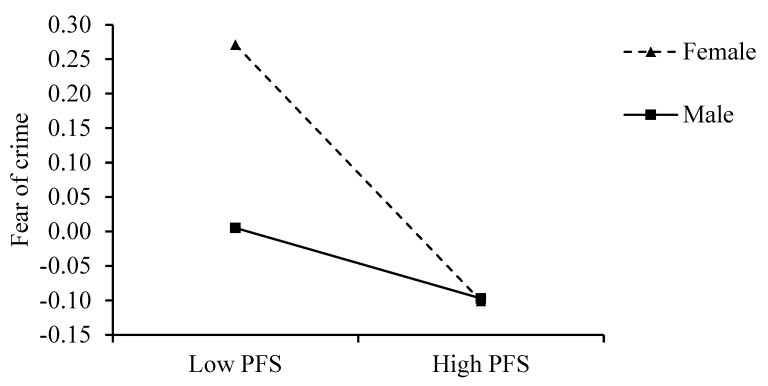
Interaction between PFS and gender on FOC.

Based on the above-mentioned mediating effect, we proposed that gender also regulated the association between family support and FOC, and then regulated the mediating effect of family support on sleep quality with respect to FOC and the chain effect of family support–FOC–loneliness–sleep quality. To evaluate this proposal, we first explored whether the impact of family support on FOC was moderated by gender (Table 3). Family support had a significant negative impact on FOC after adding gender and the interactive term of family support and gender had a significant impact on FOC, indicating that gender can moderate the impact of family support on FOC. To further explain the regulatory effect of gender, we performed a simple slopes analysis [106], as shown in Figure 2. For female respondents (gender = 0), family support had a significant negative impact on FOC (B = −0.154, *p* < 0.001, 95% CI [−0.210, −0.100]); for male respondents (gender = 1), family support had a significant impact on FOC (B = −0.046, *p* = 0.033, 95% CI [−0.088, −0.004]). We constructed an indicator to represent the difference between family support and the FOC regression coefficient for female and male respondents. The results of 10,000 bootstrap calculations showed a significant difference in the regression coefficient between female and male respondents (95% CI [0.040, 0.176], not including zero). Thus, compared with male residents, family support had a greater impact on female respondents’ FOC. Therefore, Hypothesis 5 was supported.

Given that gender can regulate the pathway through which family support affects people’s FOC, we continued to analyze whether gender moderated the impact of family support on sleep quality with respect to FOC. Based on [101,107], we calculated whether there existed a significant difference in the mediation effect of family support on sleep quality with respect to FOC between female and male respondents separately. We explored whether gender’s mediation effect was significant by showing the difference between men and women. For female respondents, the mediating effect of family support on sleep quality with respect to FOC was significant (B = 0.045, *p* = 0.001, 95% CI [0.023, 0.077]); for male respondents as well, this mediating effect was marginally significant (B = 0.013, *p* = 0.069, 95% CI [0.002, 0.032]). We then constructed an index to represent how far the mediating effect of family support on sleep quality with respect to FOC differed between female and male respondents. The 10,000 times bootstrap sampling indicated that gender could mediate the mediating effect of family support on sleep quality with respect to FOC (95% CI [0.011, 0.062], not including zero). In other words, female respondents’ family support had a greater effect on their sleep quality with respect to FOC than that of male respondents.

In addition, we calculated whether the chain mediation effect of family support on sleep quality with respect to FOC and loneliness was significantly different between female and male respondents. For female residents, this chain mediation effect was significant (B = 0.006, *p* = 0.015, 95% CI [0.002, 0.013]); for male respondents, this mediating effect was not significant (B = 0.002, *p* = 0.131, 95% CI [0.000, 0.005]). We also constructed an index to represent how far the chain mediation effect differed between female and male respondents. The 10,000 bootstrap samples showed that gender may regulate the family support chain mediation effect on sleep quality with respect to FOC and loneliness (95% CI [0.001, 0.010], not including zero).

### 3.6. Outcomes without Control Variables

Based on [108,109], we further explored whether the results of the main models changed significantly in the absence of control variables. In the direct model in which family support affected sleep quality, the impact of family support on sleep quality was still significant without control variables (B = 0.129, *p* < 0.001, 95% CI [0.056, 0.201]). In the mediation model in which family support affected sleep quality with respect to FOC, we found that if there were no mediating variables including age and education level, the mediating effect of family support on sleep quality through FOC was still significant (B = 0.029, *p* < 0.001, 95% CI [0.016, 0.048]). In the moderated mediation model, if the control variable was not added, the interactive term between family support and gender was significant (B = 0.104, *p* = 0.002, 95% CI [0.039, 0.170]); for female respondents, family support had a significant mediating effect on sleep quality with respect to FOC (B = 0.050, *p* < 0.001, 95% CI [0.028, 0.082]). Overall, we concluded that the effect of family support on sleep quality with respect to FOC was significant (B = 0.015, *p* = 0.057, 95% CI [0.002, 0.033]; at the same time, the difference between the mediating effect among female and male respondents was significant (95% CI [0.014, 0.068], not including zero). Therefore, gender moderates the mediating effect of family support on sleep quality with respect to FOC. The above results are consistent with the results of the model with control variables, indicating that the model proposed in this study has strong robustness [108].

## 4. Discussion

### 4.1. Theoretical Implications

The theoretical contributions of this study are mainly reflected in the following aspects. First, we explored several potential mechanisms through which family support affects the sleep quality of middle-aged and older residents. We found that family support can improve sleep quality by reducing residents’ FOC and by reducing their loneliness. We also found that family support can improve sleep quality through a chain mediation effect by reducing FOC, and then reducing the path of loneliness to improve people’s sleep quality. These results deepen the understanding of the association between family support and the sleep quality of middle-aged and older people whose outcomes are also coherent with the main idea from the social support model, and also provide new ideas for further intervention research on sleep quality improvement. Second, this study enhances the research on the interactive effects of family support and gender on important outcome variables which is also reflected with the research outcomes of the vulnerability hypothesis. We propose that gender mediates the effect of family support on FOC, further influencing the impact of family support on sleep quality. Our findings may increase the understanding of gender differences in family support effects and provide new evidence for gender differences in FOC and loneliness.

### 4.2. Practical Implications

First, family support may be one of the most effective ways to improve the sleep quality of middle-aged and older people. This study shows that family support can improve their sleep quality by reducing their FOC and loneliness. It is important to include the family impact factor in improving sleep quality. Family support should be included in cases when the media and other social organizations carry out campaigns related to improving sleep quality. Based on our analysis, doctors could consider family support as one the important ways to treat sleep quality issues besides giving patients appropriate medication and psychotherapy.

Second, family support affects sleep quality with respect to FOC and loneliness, while individual’s sleep quality could be improved by reducing FOC and loneliness. Therefore, people need to be aware of the importance of family support to reduce FOC and loneliness and understand the significance of family support in reducing their fear of crime and loneliness and improving sleep quality. On the other hand, this study supports the idea that reducing FOC and loneliness compensates for the effect of family support on sleep quality to a certain extent. Which means, in the case of individuals experiencing low levels of family support, they could also reduce the potential negative effects caused by insufficient family support by alleviating FOC or loneliness.

Third, it is important to pay attention to the gender differences in family support and sleep quality improvement. This study found that family support had a greater effect on women’s FOC, and this effect extended to sleep quality through the association between FOC and loneliness. Therefore, we argue that women need more care from family members [78]. Help from family members may not only reduce the emotional pressure on women caused by criminal activities, but also better improve their sleep quality.

### 4.3. Limitations

This study has certain limitations. First, this study used a cross-sectional design, limiting the possibility of examining the causal effects of FOC and loneliness on sleep quality. In the future, we need to consider the use of a longitudinal research design to further explore the intermediary role of FOC and loneliness in the relationship between family support and sleep quality based on a combined method—quantitative and qualitative—where the qualitative aspect may include interviews with some of the participants to obtain a better and deeper understanding of the effects of the research variables from the subjective point of view of the participants. Second, this study could further improve its measurement indicators. For example, more items should be included in the questionnaire. Because this study focused on middle-aged and older people, we limited the number of measurement items to prevent fatigue. Many studies have shown that short measurement scales have high reliability [97]. However, future research could consider using more extensive measurement scales with more comprehensive questions. Future research could also consider including actigraphy and other instruments to analyze residents’ objective sleep quality instead of only using self-report methods as this study does. Third, there may be multiple mechanisms through which family support affects sleep besides those proposed in this study. The impact mechanism we propose between FOC and loneliness may be important; however, this study cannot rule out the possibility of the existence of other mechanisms. Future research should continue to explore other potential mechanisms between family support and sleep quality. Future research could also continue to explore the relationship between FOC and loneliness. We propose that the FOC impacts the loneliness condition of participants, while loneliness may also impact the FOC, but other variables, such as isolation from children (or other relatives) or neighborhood socioeconomic status, may also impact on FOC and loneliness simultaneously. Fourth, future research could consider designing intervention programs based on the results of this study, and explore how to improve the sleep quality of middle-aged and older residents by improving family support. Fifth, future research could use a circular perspective to conduct sleep studies. For example, lack of family support may affect people’s sleep quality, and low-quality sleep may make residents emotionally unstable, leading to behavioral problems [110]. These problems may make it more difficult for people to obtain family support and other social support.

## 5. Conclusions

This study draws the following conclusions. Family support has a significant positive effect on the sleep quality of middle-aged and older people. FOC and loneliness are intermediate variables between family support and sleep quality, and the chain mediation effect of family support affecting loneliness with respect to FOC and then affecting sleep quality is also significant. Gender plays a moderating role in family support and FOC. Compared with men, the family support for middle-aged and older women has a greater impact on FOC, and the family support for women affects sleep quality with respect to FOC. The mediation effect is also stronger among women. For female middle-aged and older residents, the chain mediation effect of family support–FOC–loneliness–sleep quality is significant, but for male middle-aged and older people, this effect is not significant.

## Figures and Tables

**Table 1:** Means, standard deviations, and correlations for study variables.

Variable	M	SD	1	2	3	4	5	6	7	8	9
1. PFS	5.35	1.26									
2. FOC	1.60	0.66	−0.16 ***								
3. LLN	1.71	0.76	−0.24 ***	0.17 ***							
4. SQ	5.03	1.44	0.11 ***	−0.18 ***	−0.15 ***						
5. Gender	0.56	0.50	−0.01	−0.10 **	−0.02	0.02					
6. Age	64.87	11.52	0.02	−0.15 ***	0.04	0.01	0.07 *				
7. Edu	0.51	0.50	0.10 **	0.02	−0.10 **	−0.00	0.16 ***	−0.29 ***			
8. MS	0.91	0.29	0.16 ***	0.05	−0.10 **	0.05	−0.00	−0.18 ***	0.15 ***		
9. LA	0.13	0.34	−0.15 ***	−0.01	0.16 ***	−0.06	0.01	0.18 ***	−0.20 ***	−0.59 ***	
10. SES	3.08	1.18	0.15 ***	−0.01	−0.08 **	0.07*	−0.03	−0.03	0.28 ***	0.07 *	−0.08 **

Note. N = 1043; PFS = perceived family support; FOC = fear of crime; LLN = loneliness; SQ = sleep quality; Edu = educational level; MS = marital status; LA = living alone; SES = socioeconomic status; * *p* < 0.05. ** *p* < 0.01. *** *p* < 0.001.

**Table 2:** Results of chain mediation model.

Variables	B	SE	*p*	Bootstrapped 95% CI	
				LL	UL
Mediator = FOC					
PFS	−0.090	0.018	<0.001	−0.126	−0.055
Age	−0.008	0.002	<0.001	−0.012	−0.005
Edu	−0.054	0.046	0.236	−0.144	0.037
MS	0.188	0.085	0.027	0.018	0.353
LA	0.075	0.080	0.346	−0.079	0.235
SES	0.008	0.019	0.698	−0.031	0.046
Mediator = Loneliness					
PFS	−0.124	0.023	<0.001	−0.168	−0.081
FOC	0.175	0.039	<0.001	0.098	0.251
Age	0.003	0.002	0.182	−0.001	0.007
Edu	−0.020	0.050	0.694	−0.115	0.078
MS	−0.028	0.113	0.805	−0.249	0.193
LA	0.233	0.099	0.018	0.046	0.434
SES	−0.021	0.021	0.308	−0.061	0.021
Outcome variable = SQ					
PFS	0.068	0.039	0.087	−0.010	0.144
FOC	−0.294	0.071	<0.001	−0.439	−0.159
LLN	−0.229	0.064	<0.001	−0.359	−0.105
Age	−0.001	0.004	0.773	−0.009	0.007
Edu	−0.140	0.098	0.152	−0.333	0.048
MS	0.120	0.208	0.563	−0.297	0.518
LA	−0.074	0.176	0.676	−0.429	0.265
SES	0.075	0.043	0.080	−0.010	0.158
PFS → FOC → SQ	0.026	0.008	0.002	0.013	0.047
PFS → LL → SQ	0.028	0.009	0.003	0.013	0.051
PFS → FOC → LL → SQ	0.004	0.002	0.022	0.001	0.008

Note. N = 939; CI = confidence interval; LL = lower limit; UL = upper limit; PFS = perceived family support; FOC = fear of crime; LLN = loneliness; SQ = sleep quality; Edu = educational level; MS = marital status; LA = living alone; SES = socioeconomic status; PFS → FOC → SQ = PFS effect on SQ with respect to FOC; PFS → LLN → SQ = PFS effect on SQ with respect to LLN; PFS → FOC → LLN → SQ = PFS effect on SQ with respect to FOC and LLN.

**Table 3:** Results of moderated chain mediation model.

Variables	B	SE	*p*	Bootstrapped 95% CI	
				LL	UL
Mediator = FOC					
PFS	−0.154	0.028	<0.001	−0.210	−0.100
Gender	−0.131	0.045	0.003	−0.220	−0.043
PFS × Gender	0.107	0.035	0.002	0.040	0.176
Age	−0.008	0.002	<0.001	−0.012	−0.005
Edu	−0.027	0.046	0.554	−0.118	0.064
MS	0.202	0.084	0.016	0.036	0.364
LA	0.099	0.080	0.212	−0.054	0.256
SES	0.006	0.020	0.753	−0.033	0.045
Mediator = Loneliness					
PFS	−0.124	0.023	<0.001	−0.168	−0.081
FOC	0.173	0.039	<0.001	0.097	0.249
Gender	−0.033	0.048	0.492	−0.125	0.061
Age	0.003	0.002	0.159	−0.001	0.007
Edu	−0.013	0.050	0.798	−0.108	0.085
MS	−0.026	0.113	0.821	−0.246	0.194
LA	0.235	0.099	0.017	0.049	0.435
SES	−0.022	0.021	0.285	−0.063	0.020
Outcome variable = SQ					
PFS	0.068	0.040	0.085	−0.011	0.145
FOC	−0.292	0.071	<0.001	−0.437	−0.156
LLN	−0.229	0.065	<0.001	−0.358	−0.104
Gender	0.040	0.095	0.675	−0.151	0.221
Age	−0.001	0.004	0.739	−0.009	0.007
Edu	−0.148	0.099	0.137	−0.341	0.045
MS	0.118	0.208	0.572	−0.299	0.515
LA	−0.075	0.176	0.669	−0.429	0.264
SES	0.076	0.043	0.075	−0.009	0.159

Note. N = 939; CI = confidence interval; LL = lower limit; UL = upper limit; PFS = perceived family support; FOC = fear of crime; LLN = loneliness; SQ = sleep quality; Edu = educational level; MS = marital status; LA = living alone; SES = socioeconomic status.

## Data Availability

The datasets generated and/or analyzed during the current study are not publicly available. The anonymized data can be obtained from two sources. First, access to the anonymized data is available from the local government, which provided financial support for the current study. Second, the anonymized data are available from the corresponding author upon reasonable request and with the permission of Anhui Normal University in China.
